# Hemihepatectomy and Replacement of The Afferent Hepatic Blood Supply in The Dog

**DOI:** 10.1155/1989/24589

**Published:** 1989

**Authors:** Egge J. Boerma, Herman H. M. de Boer, M. Niels van der Heyde, Urbain J. G. M. van Haelst

**Affiliations:** 1 Departments of Surgery and Pathology St Radboud University Hospital Nijmegen The Netherlands; 2 St Joannes de Deo Ziekenhuis Afd.Chirurgie Velserstraat 19 Haarlem 2023 EA The Netherlands

## Abstract

Hemihepatectomy along with portal vein or hepatic artery replacement in dogs was well tolerated, but combined with replacement of both vessels it was lethal because of outflow block and shock. Total liver blood flow should be kept as high as possible during such procedures in man.


*HPB Surgery* 1989, Vol. 1, pp. 101–105
Reprints available directly from the publisher
Photocopying permitted by license only

© 1989 Harwood Academic Publishers GmbH
Printed in Great Britain

### HEMIHEPATECTOMY AND REPLACEMENT OF THE AFFERENT HEPATIC BLOOD SUPPLY IN THE DOG

### Resection of Liver and Hepatoduodenal Ligament

EGGE J. BOERMA*, HERMAN H.M. DE BOER,
M. NIELS VAN DER HEYDE and URBAIN J.G.M. VAN HAELST

*Departments of Surgery and Pathology, St Radboud University Hospital, Nijmegen, The Netherlands*

*(Received 23 March 1988)*

Hemihepatectomy along with portal vein or hepatic artery replacement in dogs was well tolerated, but combined with replacement of both vessels it was lethal because of outflow block and shock. Total liver blood flow should be kept as high as possible during such procedures in man.

KEY WORDS: Bile-duct cancer, liver resection, portal vein bypass, hepatic artery replacement, liver blood flow, sleeve anastomosis.

### INTRODUCTION

Local tumor recurrence is common after resection of proximal bile-duct cancer, even when the resection is extended to a liver resection [1,2]. Simultaneous resection of the hepatoduodenal ligament with its potential extraductal and lymphatic [3] tumor spread may provide better results. Hemihepatectomy with replacement of both portal venous and arterial hepatic inflow tracts was therefore performed in dogs to examine the possibilities of liver resection with wide excision of the portal vein and the hepatic artery.

### METHODS

Thirty-five operations were performed in 17 dogs (beagles, 8–13 kg). Pressure recordings and angiography of the arterial and portal venous systems were performed, and anastomoses and liver specimens were examined microscopically.

A left hemihepatectomy was performed in all dogs. Replacement of the portal venous circulation to the retained right hemiliver was established by a portal vein bypassing conduit, formed by the splenic vein anastomosed end-to-end with interrupted sutures to a hepatic portal vein branch [4]. Replacement of the arterial inflow was carried out be transection of the hepatic and splenic arterios, followed by insertion of the central part of the splenic artery into the distal part of the hepatic artery, with the use of interrupted stitches. Such sleeve anastomosis, reported to be a fast and effective anastomosis in small vessels [5], was used to investigate its possible advantage for clinical application.

*Correspondence and requests for reprints to Dr. E.J. Boerma, St Joannes de Deo Ziekenhuis, Afd.Chirurgie, Velserstraat 19, 2023 EA Haarlem, The Netherlands.

The 17 dogs were divided into three groups according to the type of operation performed:

Group I (nine dogs): left hemihepatectomy and reconstruction of the portal venous circulation. One month later: reconstruction of the arterial hepatic inflow. One month later: exploration and sacrifice.

Group II (two dogs): left hemihepatectomy and arterial reconstruction. One month later: exploration and sacrifice.

Group III (six dogs): left hemihepatectomy and simultaneous portal venous and arterial reconstruction in one operation (Figure 1).

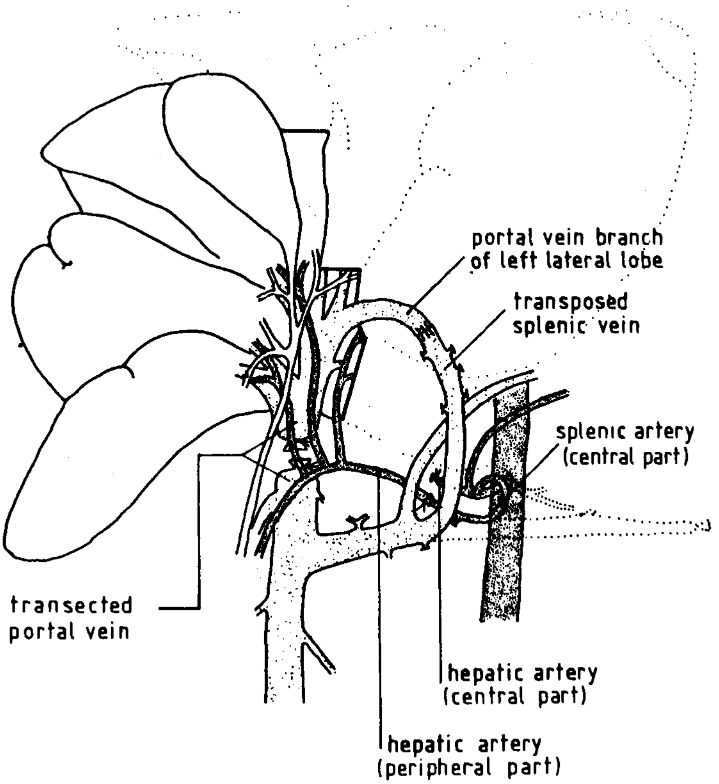

**Figure 1** Left hemihepatectomy with replacement of the afferent hepatic blood supply in the dog. The portal vein is replaced by a peripheral splenoportal bypass. The splenic artery takes the place of the central part of the hepatic artery.

### RESULTS

### *Group I*

Left hemihepatectomy with replacement of the hepatic portal venous inflow tract was carried out without problems. One month later the splenoportal anastomoses had widened to a large vessel and the livers had regenerated normally [4]. The arterial reconstruction required interruption of the hepatic arterial circulation for a mean of 16 min (range 10–32). The splenic artery (ø 1.7 mm) did not fit accurately into the hepatic artery, and an inevitable stenosing anastomosis resulted. Arterial pressures did not change.

Two dogs died in the second week after arterial replacement. Both had a blocked arterial anastomosis and venous liver congestion, indicating liver ischemia. Two dogs were in bad condition during the first week after arterial reconstruction but recovered. At re-operation they appeared to have a blocked arterial anastomosis and a collateral circulation from the left gastric artery. Five dogs withstood the arterial replacement without problems and at sacrifice they had patent but narrow anastomoses. The cut end of the inserted splenic artery tapered off into the hepatic artery by an organized thrombus covered with intimal lining.

### *Group II*

Both dogs had no postoperative problems. At re-operation their anastomoses were patent but narrow and their livers had regenerated normally.

### *Group III*

Blood loss (mean 270 $cm^3$) and rise of portal blood pressure (mean from 6 mmHg to 15 mmHg) did not differ from the levels measured at the other operations. In two dogs the arterial pressure slowly decreased after arterial reconstruction but no operative abnormalities could be found. Four dogs were quite well during the first postoperative hours. All six dogs died in shock within 24 h. Postmortem examinations revealed ascites (mean 200 $cm^3$), patent anastomoses and congestive livers with acute pericentral congestion and necrosis as seen in outflow block and shock (Figure 2).

### DISCUSSION

Vascular reconstruction has proven to be practicable in hepatic transplantation surgery. Both portal venous and arterial hepatic blood supplies have to be reconstructed for optimal hepatic function after major liver resection and wide excision of the portal vein and hepatic artery, also in clinical resection of hilar cholangiocarcinoma.

Hemihepatectomy and replacement of the portal vein by a spenoportal bypass or replacement of the hepatic artery by a splenohepatic conduit was well tolerated in dogs. Hepatic arterial transfer could also be executed satisfactory in dogs that had recovered from hemihepatectomy and portal vein replacement, provided the arterial anastomosis remained patent. The sleeve anastomosis technique had no advantages: it was not easier, it took no less time, it resulted in a narrow lumen, it did not expand, and it carried the risk of thrombosis.

Hemihepatectomy and simultaneous replacement of the portal venous and arterial hepatic blood supply was lethal. The temporarily decreased hepatic portal blood flow through the initially spastic splenoportal bypass would have caused a reactive increase of the blood flow in an intact canine hepatic artery [6,7]. Instead, the arterial hepatic blood flow was decreased by the narrow splenohepatic conduit. The sudden decline of both portal venous and arterial hepatic blood flows caused

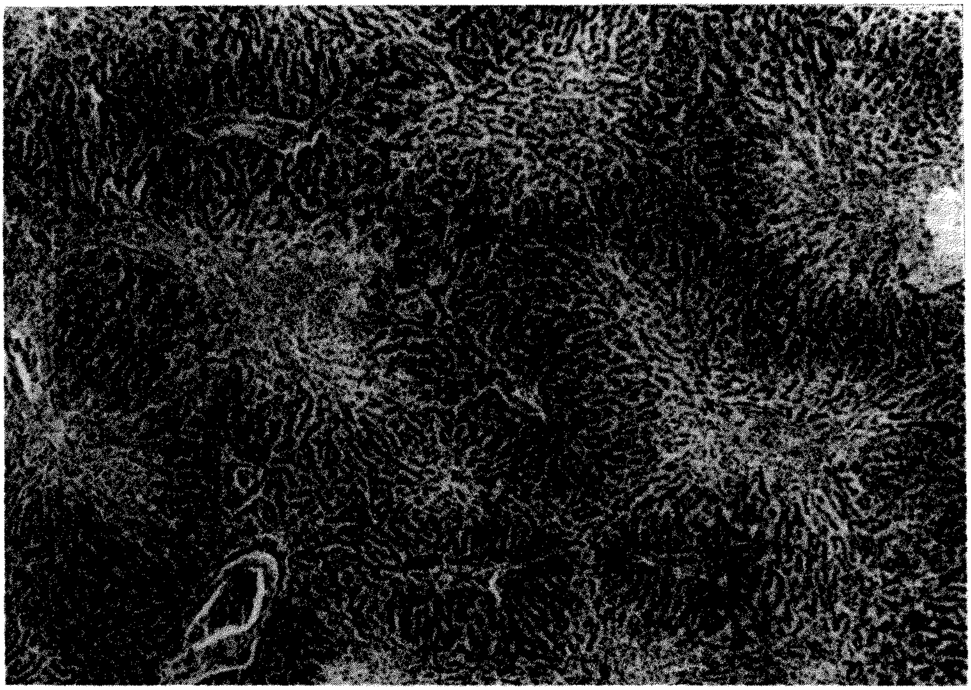

**Figure 2** Section of the liver of a group III dog that died after progressive shock: centrolobular pale areas of necrosis, hemorrhage and congestion, which often merge together; plates of normal liver cells can still be recognized around the portal tracts (HE = 60).

liver ischemia serious enough to activate the typically canine hepatic venous sphincters, thereby producing blockage of the hepatic venous outflow, and shock[8-10].

No such outflow block exists in man or pig: nevertheless, these experimental findings stress the need to preserve the total liver blood flow at as high a level as possible in human practice. This requires the use of the largest suitable vessels and wide, potentially expandable anastomoses. Interposition of a wide autologous vein graft between the splenic vein and hepatic portal vein branch to replace the portal vein would be easier in man than mobilization of the splenic vein. A saphenous interposition graft or mobilized splenic [11] or gastroduodenal artery could serve as substitutes for the human hepatic artery.

### References

1. Beazley, R. M., Hadjis, N., Benjamin, I. S. and Blumgart, L. H. (1984) Clinicopathological aspects of high bile duct cancer. *Ann. Surg.*, **199**, 623–636.
2. Tsuzuki, T., Ogata, Y., Iida, S. *et al.* (1983) Carcinoma of the bifurcation of the hepatic ducts. *Arch. Surg.*, **118**, 1147–1151.
3. Hardy, K.J., Wheatly, I.C., Anderson, A.I.E. and Bond, R.J. (1976) The lymph nodes of the porta hepatis. *Surg. Gynecol. Obstet.*, **143**, 225–228.
4. Boerma, E.J., de Boer, H.H.M. and van Haelst, U.J.G.M. (1985) Hemihepatectomy and transfer of the portal inflow tract of the retained hemi-liver in the dog. *Surgery*, **97**, 591–595.
5. Lauritzen, J., Johansson, B.R. and Erikson, E. (1980) Long-term study of the microvascular sleeve anastomosis. *Scand. J. Plast. Reconstr. Surg.*, **14**, 165–169.

6. Kock, N.G., Hahnloser, R., Roding, B. and Schenk, W.G. (1972) Interaction between portal venous and hepatic arterial blood flow: an experimental study in the dog. *Surgery,* **72**, 414–419.
7. Mathie, R.T., Lam, P.H.M., Harper, A.M. and Blumgart, L.H. (1980) The hepatic arterial blood flow response to portal vein occlusion in the dog. *Pflügers Arch,* **386**, 77–83.
8. Neill, S.A., Gaisford, W.D. and Zuidema, G.D. (1963) A comparative study of the hepatic veins in the dog, monkey and human. *Surg. Gynecol. Obstet,* **116**, 451–456.
9. Starzl, T.E., Kaupp, H.A., Brock, D.R. *et al.* (1960) Reconstructive problems in canine liver homotransplantation with special reference to the postoperative role of hepatic venous flow. *Surg. Gynecol. Obstet.* **111**, 733–743.
10. Walker, W.F., MacDonald, J.S. and Pickard, C. (1960) Hepatic vein sphincter mechanism in the dog. *Br. J. Surg,* **48**, 218–220.
11. Longmire Jr, W.P., McArthur, M.S., Bastounis, E.A. and Hiatt, J. (1973) Carcinoma of the extra-hepatic biliary tract. *Ann. Surg.* **178**, 333–345.

*Accepted by L Blumgart on 23 March 1988.*

